# A quantitative estimation of the global translational activity in logarithmically growing yeast cells

**DOI:** 10.1186/1752-0509-2-87

**Published:** 2008-10-16

**Authors:** Tobias von der Haar

**Affiliations:** 1 Protein Science Group, Department of Biosciences, University of Kent, Canterbury, CT2 7NJ, UK

## Abstract

**Background:**

Translation of messenger mRNAs makes significant contributions to the control of gene expression in all eukaryotes. Because translational control often involves fractional changes in translational activity, good quantitative descriptions of translational activity will be required to achieve a comprehensive understanding of this aspect of biology. Data on translational activity are difficult to generate experimentally under physiological conditions, however, translational activity as a parameter is in principle accessible through published genome-wide datasets.

**Results:**

An examination of the accuracy of genome-wide expression datasets generated for *Saccharomyces cerevisiae *shows that the available datasets suffer from large random errors within studies as well as systematic shifts in reported values between studies, which make predictions of translational activity at the level of individual genes relatively inaccurate. In contrast, predictions of cell-wide translational activity are possible from such datasets with higher accuracy, and current datasets predict a production rate of about 13,000 proteins per haploid cell per second under fast growth conditions. This prediction is shown to be consistent with independently derived kinetic information on nucleotide exchange reactions that occur during translation, and on the ribosomal content of yeast cells.

**Conclusion:**

This study highlights some of the limitations in published genome-wide expression datasets, but also demonstrates a novel use for such datasets in examining global properties of cells. The global translational activity of yeast cells predicted in this study is a useful benchmark against which biochemical data on individual translation factor activities can be interpreted.

## Background

The rate of translation of eukaryotic messenger RNAs is often stringently controlled and can limit the overall amount of protein produced from a gene. Moreover, changes in mRNA-specific translation rates can occur, and such changes can alter protein production rates independently of transcriptional activity. Translational control mechanisms of this kind are known to contribute to cellular growth and proliferation, the development of organisms, and to the occurrence of disease [1].

Mechanisms by which the translational apparatus can exert control over gene expression rates are being described in increasing detail. For simple organisms like yeast it is now likely that we know most of the canonical translation factors, and many or most of the non-canonical factors that exert mRNA-specific effects [2]. Nevertheless, the detailed modes of translational control remain elusive, and even where factors are well studied effects of small alterations in their activity are still impossible to predict quantitatively.

Most of our difficulties in interpreting translational control in real life likely stem from the fact that the mechanisms in question do not employ simple on-off switches, but instead use fractional changes in the activity of their component parts to achieve redistributions of translational activity among different mRNA species. Such fractional changes cannot be understood from the structure of pathways alone, but additionally require quantitative knowledge of reaction rates, fluxes, etc. The development of reliable quantitative models of translation is thus an important prerequisite for the understanding of gene expression.

More and more aspects of the translational pathways in yeast are being examined in quantitative detail, including assembly of the cap-binding complex [3], formation of the ternary eIF2:GTP:tRNA_i_^Met ^complex [4,5], formation of the 43S complex [6], ribosomal subunit joining [7], and many aspects of translation elongation [8,9]. The results from these studies already form an important basis for an increased quantitative understanding of eukaryotic gene expression. However, scope for the interpretation of results from such work is limited because to date we do not have a comparative value of how much gene expression activity yeast actually requires. The relevant parameters (proteins produced and amino acid bonds formed per unit time) are difficult to determine experimentally and, where relevant techniques exist at all, these techniques require the application of specific and often non-physiological growth conditions. The principal approach for the experimental determination of translation rates is based on measurements of incorporation rates of isotopically labelled amino acids, which can only be performed in defined (minimal) medium and usually involves limiting amounts of the labelled amino acid. Moreover, while this technique is routinely used for the estimation of *relative *changes in translation rates (see e.g. refs [10-12] for representative examples), the author is not aware of any reports of *absolute *estimates for cell-wide translation rates that have to date been reported using this or any other experimental approach.

Rate information on translational activity should also be represented in the genome-wide protein abundance and half-life surveys that have been published over the last years [13-16]. Indeed, such information has been extracted in the past based on individual genes [17,18]. However, comparisons between overlapping portions of datasets from independently conducted studies suggest that there is a considerable error associated with genome-wide abundance data [15], and it is not clear in how far the accuracy of rate-information derived from error-prone data is itself affected by error. In this study, genome-wide datasets are examined with respect to the likely extent of error that they contain, and it is shown that global rate information is likely to be more accurately represented than rate information for individual gene products. Global translation rates predicted from the currently available datasets are then shown to fit relatively well with a number of completely independently derived parameters, including the cellular active ribosome content, and kinetic models of various nucleotide exchange reactions that occur during translation.

## Methods

### Raw datasets

Reported protein abundance data were retrieved from the supplemental material accompanying published studies by Ghaemmaghami *et al*. [14], Newman *et al*. [16] and Lu *et al*. [15]. Only datasets obtained with yeast strains grown in rich medium (YPD) were used. An additional dataset based on abundance values reported in individual studies in the published literature was constructed via full-text searches of the relevant literature on Textpresso  and Google Scholar  (see Additional file 1 for details of this dataset). Transcriptome data reporting absolute mRNA abundances for YPD-grown yeast were retrieved from studies published by Holstege *et al*. [19], Arava *et al*. [20], Holland [21], Jelinsky and Samson [22] and Roth *et al*. [23]. Genome-wide protein half-life values for growth in YPD were retrieved from the publication by Belle *et al*. [13]. A second protein half-life dataset was constructed by extracting 202 protein half-life values from small-scale studies (Additional file 1).

### Pairwise study comparisons

For each pairwise comparison between two datasets, genes for which non-zero values existed in both datasets were extracted, and for each gene the fold difference in reported values was calculated. These fold difference values were then log_10_-transformed, and distributions of the log_10 _fold difference values were analysed using the dfittool function in the Matlab statistics toolbox (release 2007b, The Mathworks Inc., Natick, MA). Histograms of the log fold difference values were constructed using the Freedman-Diaconis rule, and normal distributions were fitted to the resulting histograms.

### Construction of a curated dataset

Information from the genome-wide studies was collated into a curated dataset as follows: information from the pairwise comparisons was initially used to scale the protein and mRNA abundance datasets to a common mean (scaling factors for the protein abundance studies were Ghaemmaghami *et al*. = 0.84, Lu *et al*. = 0.91, Newman *et al*. = 12.6, Literature set = 0.61; for mRNA abundance studies Holstege *et al*.= 1.2, Arava *et al*. = 1.8, Holland = 0.7, Jelinsky and Samson = 0.929, Roth = 0.3). Where available, experimental data from the genome-wide studies were then imported into the curated dataset.

In order to enrich the dataset for missing values, all genes without an assigned protein abundance value were then analysed using data from the Ghaemmaghami *et al*. and Newman *et al*. studies[14,16] to identify those genes which had been successfully tagged with both GFP and TAP-tags, but where expression of neither tag could be detected. These genes were assigned an abundance value of 0. For all other genes, missing values on protein abundance and protein turnover were assigned by using mRNA abundance value as a predictor for protein abundance (based on an average of 3450 proteins per mRNA for all genes where both values were determined experimentally), and by using the average protein half-life of all proteins with experimentally determined values as a "best guess" for those genes where no turnover information was available (half-life of 778 minutes or decay-rate constant of 0.00089 min^-1^). The final curated dataset is given in Additional file 1, with individual entries colour-coded to identify their mode of construction.

### Calculation of global gene expression parameters

The translational frequency *F*_*g *_for an individual gene *g *was calculated as:

*F*_*g *_= *A*_g _* *k*_*g *_+ *A*_g _* *k*_*Growth*_,

where *A*_*g *_is steady-state protein abundance for gene *g*, *k*_*g *_is the gene-specific protein decay rate constant calculated from the observed half-lives as *k*_*g *_= *ln(2)/t*_1/2,*g*_, and *k*_*Growth *_is the global apparent dilution rate constant resulting from cell growth, calculated from the doubling time of 90 minutes as *k*_*Growth *_= *ln(2)/90*. *F*_*g *_represents the rate of protein production per minute for gene *g*, and equals frequencies of translation initiation and translation termination if 100% efficiency of these processes and no "drop-off" of ribosomes during translation elongation are assumed. Translation elongation frequencies *E*_*g *_for individual genes were calculated as:

*E*_*g *_= *F*_*g *_× *(L*_*ORF*,*g *_- *1)*,

where *L*_*ORF*,*g *_is the ORF length of gene *g *in codons. Global frequencies were calculated as the sums of *F *and *E *for all genes.

### Analysis of ribosome densities

The number of ribosomes involved in the synthesis of protein from an individual gene *g *at any given time, *R*_*g*_, was calculated as:

*R*_*g *_= *F*_*g *_× *L*_*ORF*,*g*_/*ε*

where *ε *is the average translation elongation rate in codons per second. The sum of *R *for all genes was used to estimate total numbers of active ribosomes per cell for a given *ε*.

### Modelling of nucleotide exchange

Catalysed guanidine nucleotide exchange rates on eIF2 were computed using the Michaelis-Menten equation from the published *K*_*M *_and *K*_*cat *_values [5]. A unidirectional reaction of the form

eIF2:GDP + GTP -> eIF2:GTP + GDP

was assumed, reflecting the assumption that eIF2:GTP complexes were withdrawn from the system immediately upon formation and fed back as eIF2:GDP complexes, which allowed calculation of the maximum rates of exchange that could be maintained at a given combination of eIF2B and eIF2:GDP concentrations.

All other nucleotide exchange reactions were implemented as kinetic models in Berkely Madonna software (v8, ) based on published kinetic information (see main text for details). Models were implemented using starting concentrations of 0.13 mM for GDP, 1.3 mM for GTP [10,11], and varying concentrations of factor:GDP complexes. The starting concentration of free factor and factor:GTP complexes was set to zero. Simulations were run until the systems reached equilibrium, and the maximum rate of factor:GTP complex formation that occurred on the way to equilibrium was recorded as a function of initial factor: GDP complex concentrations.

## Results and discussion

### Comparison of genome-wide datasets

The minimal information required to determine translational activity comprises protein abundance and protein turnover rates. In addition, mRNA abundance data can be used to analyse mRNA-specific translational activity. For budding yeast, large-scale studies have been published that report such data for closely related S288C-derived strains and essentially identical growth conditions (i.e. logarithmic growth in YPD) [13-16,19-23]. For protein and transcript abundance, these studies report partially overlapping results sets. Because of the similarity in growth conditions, these overlapping results sets should report identical data in the absence of any error; conversely, differences in reported values can be usefully employed to estimate the error that is actually associated with the datasets. In order to increase the scope for such inter-study comparisons, additional, less extensive datasets on protein abundance and half-lives were created by examining the published literature for smaller-scale studies that reported relevant values (see Additional file 1 for these datasets).

**Figure 1:**
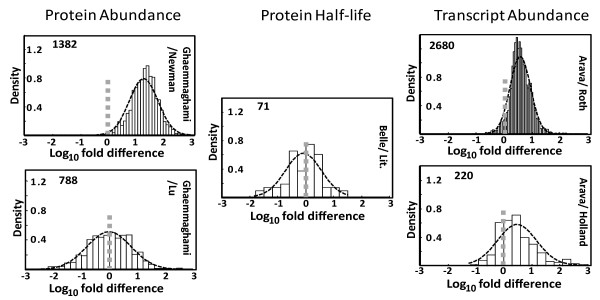
**Pair wise comparisons of genome-wide data sets**. Datasets were compared in a pair wise manner, and fold difference values were calculated for all genes that were represented in both studies of each comparison. The comparisons with the lowest (top) and highest (bottom) variance in the log difference values are shown. For protein half-life data, only a single genome-wide study is available and these data were therefore compared to protein half-life values extracted from published individual studies. In all graphs, observed log_10 _fold difference values are displayed as bars, and the best fitting lognormal distribution is displayed as a broken line. Inset numbers in the graphs indicate the number of data points (n), and grey broken lines indicate the expected pair wise comparison value for genes where two studies report the same abundance or half-life (i.e. a fold-difference value of 1 or 10^0^).

In order to estimate the reliability of data contained in individual datasets, studies addressing the same parameter (protein abundance, protein half-life, or mRNA abundance) were arranged in all possible pair wise combinations, genes for which data were reported in both datasets of the pair were extracted, and the fold difference of reported values was calculated for each gene and then log-transformed. Figure 1 shows some of the distributions of the log_10 _fold difference values resulting from these analyses. Importantly, the results clearly highlight that for most of the genes where data are reported in more than one study, these values differ widely, and thus demonstrate that at least some of the studies are associated with a relatively large error. The shape of the log fold difference distributions also indicates that there are likely to be two independent sources of error: first, for all pair wise analyses conducted in this way, the fold difference values appear to be lognormally distributed with high probability, which is most consistent with a random error within each dataset where over- or under-estimation by a given factor are equally likely. The relative magnitude of this error becomes apparent in the spread of the log-normal distributions. In additional analyses, no significant correlations could be detected between log_10 _fold difference values for individual gene products and other parameters like molecular weight and overall abundance (data not shown).

Second, there appear to be systematic shifts in reported values between studies. For example, for those proteins for which abundance values are reported in both the studies by Ghaemmaghami *et al*. [14] and Newman *et al*. [16], these values are on average about 16-fold higher in the former study. This type of error becomes apparent in the shift of the mean of the relevant distributions away from zero. Importantly, this combination of errors is observed in all the analysed studies, irrespective of whether they report data on protein abundance, protein half-life, or mRNA abundance.

### Analysis of ribosomal protein abundances

In order to validate the conclusions from the pair-wise dataset analyses, the distribution of ribosomal protein (RP) abundance values in the study by Lu *et al*. [15] was examined as an independent approach. RPs should in principle form a useful standard for protein abundance data, since there is strong evidence that these proteins occur at one copy per ribosome within assembled ribosomes (reviewed in ref. [24]), but do not generally exist in ribosome-free forms. Indeed, non-ribosome incorporated RPs tend to be rapidly degraded [16,17], so that it can be expected that the intracellular ratio of the majority of RPs is close to one.

However, the analysis of RP abundances is complicated for two of the protein abundance studies, which used C-terminal protein tagging with either GFP [16] or TAP-tags [14] for the quantification procedure. Because of the dense packing of RPs within the ribosome, the introduction of large tags is likely to interfere with RP incorporation and potentially alter turnover rates for the tagged proteins, thus reducing the apparent RP abundance. This would introduce a specifically larger error into RP abundance values compared to the rest of the proteome, and analyses of RP abundance should therefore not be used to estimate overall errors in these two studies. Indeed, the two protein abundance datasets that used tagging approaches show a specific underrepresentation of ribosomal proteins when compared to the mass-spectrometry based study by Lu *et al*. which did not rely on protein tags (data not shown).

**Figure 2:**
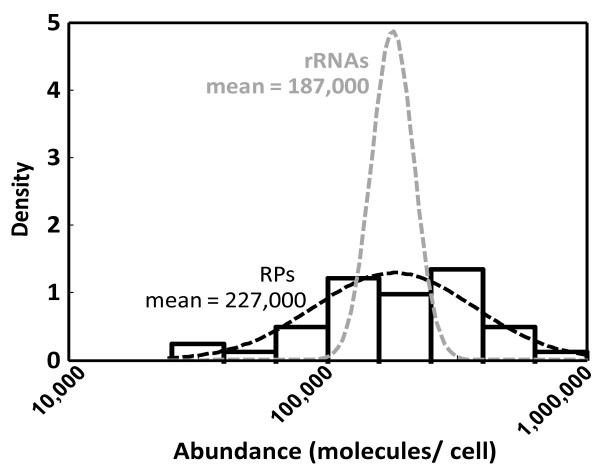
**Comparison of reported ribosomal protein and ribosomal RNA abundances**. Abundance values for 41 ribosomal proteins (rPs, black bars) were extracted from the mass spectrometry-based study by Lu *et al*. [15]. Where one rP is encoded by two genes, total rP abundance was calculated as the sum of the abundances associated with each gene. The best fitting lognormal distribution is shown as a black broken line. The grey broken line shows the distribution of independently derived rRNA abundance values.

This latter study contains 41 RPs for which complete abundance information is available, i.e. either RPs encoded by one gene with an associated value, or RPs encoded by two genes that both have an associated value. Reported abundances for these RPs range from 29,000 to 710,000 proteins per cell (figure 2). Importantly, RP abundance values appear to vary around a mean in a lognormal distribution, similar to the distributions of fold difference values discussed above. Thus, ribosomal protein abundance values in this particular dataset do not show the close distribution around a mean that is suggested by the literature for true ribosomal protein abundances, but rather show a broad, lognormal distribution as would be expected from the assumption that each dataset contains significant random error. A comparison of the variance observed for ribosomal protein abundance values in the dataset by Lu *et al*. with the variance observed for the pairwise comparison of the Ghaemmaghami *et al*. and Lu *et al*. studies, suggests that it is likely that errors in both studies contribute to the differences observed between them. However, because these variance values were generated with different datasets, they cannot be used to characterise the average error within each study more quantitatively.

The RP abundance distribution in figure 2 shows a mean abundance of 227,000 RPs per cell. Independent estimates for the cellular ribosome content have been generated by analysing the abundance of ribosomal RNA (rRNA) species in several studies [25-30], with reported values ranging from 150,000 to 350,000 copies of ribosomal RNA per cell for fast-growing haploid yeast strains. All of these studies were relying on relatively inaccurate estimates for the molecular weights of rRNAs derived from gel electrophoreses, and much of the variation in reported rRNA abundances derives from the fact that different estimates for this parameter were used. However, these studies also report the raw data for total cellular RNA content and the proportion of RNA that is rRNA, and rRNA abundances are therefore here re-calculated from these raw data based on the exact rRNA molecular weights from now available sequence information (table 1). The resulting estimate of 187,000 ± 56,000 rRNA copies per yeast cell can be usefully compared to the distribution of RP abundances in figure 2. Overall, the analysis of ribosomal protein abundance data thus supports the assumption generated from the pairwise study comparisons that individual datasets contain random errors in the reported values.

**Table 1:** Cellular RNA content and proportion of total RNA that is rRNA from several studies, and calculation of the cellular ribosome content.

**Total RNA content (g/cell)**	**rRNA proportion in total RNA**	**Ribosomes (molecules/cell) **^**a**^	**Study**
7.6 × 10^-13^	80%		[27]
4.9 × 10^-13^	84%		[30]
5.8 × 10^-13^	83%		[25]
7.0 × 10^-13^			[29]
10.0 × 10^-13^			[28]
	85%		[26]
7.1 ± 1.9 × 10^-13^	83 ± 2%	187,000 ± 56,000	

The last row contains averages.

^a ^calculated as (total RNA * rRNA proportion)/MW*N_A_; where MW is the total molecular weight of ribosomal RNA contained within a ribosome (1.9 × 10^6 ^g/mol, from ), and N_A _is Avogadro's number (6.022 × 10^23 ^mol^-1^).

### Extent of systematic errors

**Figure 3:**
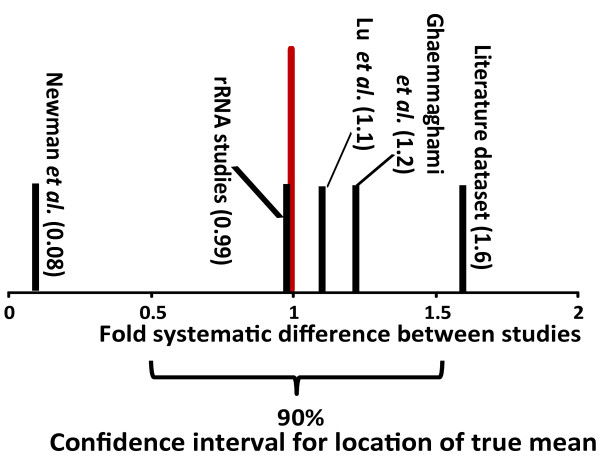
**Systematic shifts in reported values between protein abundance studies**. Bars indicate the position of studies relative to each other, which was determined by analysing the locations of means from pair wise study comparisons as shown in figure 1. The scale is normalised to the mean location of the differences between studies (indicated by a red bar). The 90% confidence interval for the location of the true mean, corresponding to the point to which the studies would need to be scaled in order to correct for the systematic errors, is indicated below the graph.

The fact that the expected values of the lognormal distributions in figure 1 are significantly different from zero indicates that, in addition to random errors, there is a second error element at least in some studies that systematically shifts values in the affected datasets. In the following, this second error element will be referred to as the location error, since it shifts the location of the log mean of fold difference values away from zero. The relative location of values reported in the three different large-scale studies and the literature dataset on protein abundance is shown in figure 3, and was derived from information including all possible pair-wise comparisons between these studies. rRNA abundance studies are included in this analysis via their comparison with the Lu *et al*. RP values. A separate analysis of location errors in the mRNA abundance datasets shows that these datasets show a similar range in systematic shifts as the protein abundance datasets. For protein half-lives, the two available datasets (the study by Belle *et al*. and the literature dataset) show only minor systematic shifts, although it is difficult to judge the statistical significance of this.

If the magnitude of the location error was exactly known for each study, then individual datasets could easily be scaled to correct for this error. Under the assumption that the location error in different studies is normally distributed, the most likely position for the true location they should have without error corresponds simply to the mean of the observed locations. This location is indicated in red in figure 3 (note that the axis was scaled relative to this point). However, because of the large shifts between studies and the small number of studies available, this estimate is inaccurate, and the location of the true mean can only be pinpointed to a range of about 50% above and below the observed mean at the 90% confidence level.

### Use of data sets for parameter prediction

Based on the types and extent of errors in the genome-wide datasets identified above, it is possible to predict the accuracy of parameters calculated from these datasets. One such parameter that is of general interest is the mRNA-specific translation rate, which follows directly from protein levels and decay-rates (to give the total translation rate for an individual gene product), and mRNA levels (to give the mRNA-specific translation rate). It is clear that the result of any calculation performed with three parameters that have a significant associated error will itself have an associated error, and an important question is thus whether the error in the results will still permit the drawing of relevant conclusions. In terms of mRNA-specific translational activity, a frequently asked question is whether two mRNAs are translated at different rates, which would be indicative of a translational control mechanism impinging on at least one of the two mRNAs.

**Figure 4:**
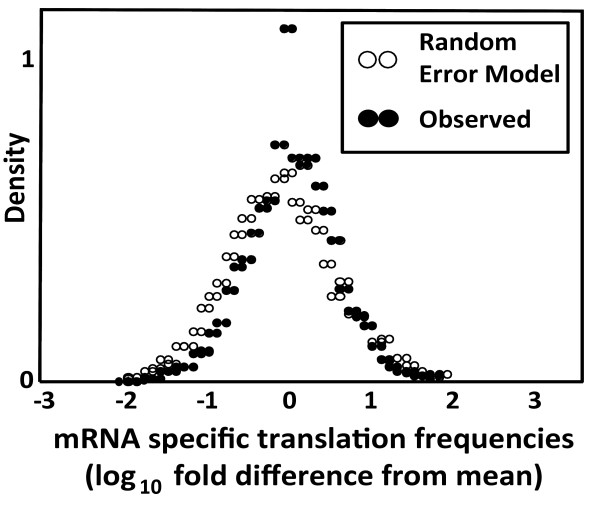
**Accuracy of parameter estimates from genome-wide studies**. Apparent translational frequencies per mRNA were calculated from protein abundance, protein half-life, and mRNA abundance values, and the resulting datasets were analysed for the variation around the mean translational frequency. The distribution outlined by open circles was generated from simulated datasets, and corresponds to the expected distribution if true translational activity was the same for all messages, but was calculated from error-prone datasets. Black circles represent the variation around the mean translational frequency for the real datasets (average values were used where more than one study reported relevant values for a gene).

In order to empirically estimate in how far the random errors contained in genome wide datasets would affect calculations of relative mRNA specific translational activity values, a simulated dataset of 4000 genes was constructed with associated protein abundance, protein half-life, and mRNA abundance values. Expression levels and half-lives in this dataset were assumed to be identical for all entries, but were allowed to vary randomly around the true value in a lognormal distribution with variances similar to those estimated for the observed error in the real genome-wide datasets. This dataset can thus serve as an approximate reference for the kind of spread in apparent mRNA-specific translation efficiencies that is falsely generated by noise in the underlying studies. The distribution of calculated mRNA specific translation frequencies that would be expected if all frequencies were identical in reality, but were calculated from noisy datasets, is shown in the histogram in figure 4 in open circles. Importantly, mRNA-specific translation frequencies calculated from the real datasets (full circles in this figure) show a very similar distribution around the mean. Thus, even for mRNAs at the extremes of the translation frequency distribution which show apparent translational rates that differ by four orders of magnitude, this difference is more likely to arise from errors in the underlying datasets than from true biological differences. This effect is caused entirely by the random error within the studies, in addition, the systematic location error between studies will affect the accuracy of calculations of absolute translation rates per mRNA.

Although this analysis highlights the problems with interpreting *local *(i.e. gene-specific) parameters calculated from the genome-wide datasets, the main aim of the study presented here is to ask whether the datasets would allow the prediction of *global *translational parameters with any accuracy. There are important implications in the observation that a large part of the variability observed in comparisons between studies appears to be random. For the calculation of global parameters, where essentially the sum of all entries in a dataset is considered, random errors within studies will be largely lost through averaging. Analyses of simulated datasets with properties similar to the experimental abundance studies indicate that the remaining error for a calculation of the global protein content is reduced by >>99% compared to the average error in the reported abundance for an individual gene product. For global parameters, the positioning error associated with the datasets is thus predicted to be the only significant error source that remains.

### Estimation of cell-wide translational activity

**Figure 5:**
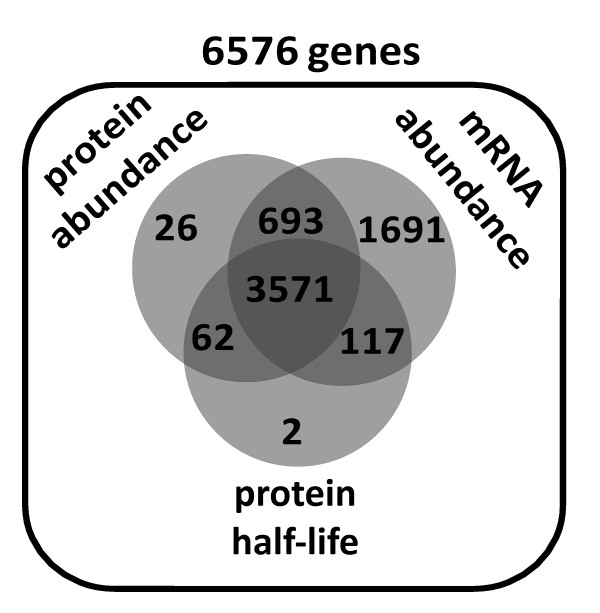
**Composition of the modelling dataset**. The diagram shows the numbers of genes for which experimentally determined mRNA abundances, protein abundances, and protein half-lives are present in the curated dataset. Note that the total set of 6576 genes includes 812 genes classed as dubious in SGD , most of which have no associated data in the curated dataset.

An analysis of the coverage of genome-wide abundance datasets (figure 5) indicates that more than half of all genes (~54%) now have associated experimental data for protein abundance, protein half-life, and mRNA abundance. A small proportion (~13%) has only one of these parameters missing. In contrast, for a relatively large proportion (~25%) mRNA values have been reported but no protein abundance or half-life values exist. Although mRNA levels for these genes have been reported, these are significantly lower than for those genes where protein abundance levels exist. Indeed, 50% of genes without existing protein abundance values have reported steady-state mRNA expression levels of less than 0.01 copies per cell. Since comparisons of real-time PCR data with microarray analyses have shown that the latter tend to overestimate very low abundance mRNAs [21], these genes are likely to be largely transcriptionally repressed. In contrast, only about 5% of genes that do have associated protein expression values have reported mRNA expression values in this low range.

Further support for the assumption that a large proportion of genes without reported protein abundance values are genuinely non-expressed comes from the two genome-wide tagging studies [14,16]. Genes that have no associated protein abundance values in these two studies fall into either of two classes: those where successful tagging could be performed and verified by genetic methods but where the tag nevertheless remained undetectable, and those were tagging could not be successfully performed. The inability to visualise either GFP- or TAP-tagged versions of the same protein where both tags could be successfully integrated is strong evidence for low or no expression at the protein level, and this criterion applies to 64% of genes without any associated protein abundance value. In conclusion, although a certain proportion of genes without such values will undoubtedly turn out to be false negatives, it can be predicted that the currently available genome-wide data sets capture the majority of expressed genes.

Based on these analyses, data from the individual genome-wide studies were pulled together into a curated dataset. The procedure for the construction of this dataset was designed to incorporate the best available experimental data wherever such data were available, and to use best guesses where such data were unavailable. Details of the construction of this dataset and the dataset itself are given in the Methods section above, and in Additional file 1.

Two global parameters that can easily be calculated from this dataset are total protein and mRNA content, and for both experimental estimates are available for comparison. The total cellular protein content reflected in the curated dataset amounts to 3.72 pg per cell, and 90% confidence limits can be estimated for this value of 1.9 and 5.6 pg based on the extent of the positioning error for protein abundance datasets. This compares to experimental estimates generated by us in two independent studies, which gave a total cellular protein content of 5 pg [12,31]. Although the total *mass *of cellular RNA cannot be calculated in the same way because non-coding mRNA regions for each gene contribute to the molecular weight but are not accurately known, the total *number *of mRNAs in the dataset can easily be calculated as about 12,200, with 95% confidence limits between 6,100 and 18,300 mRNAs per cell. This compares to experimental estimates of about 15,000 poly(A) tailed RNAs per cell generated experimentally [32]. In two important aspects, the curated dataset thus approaches estimates from available experimental data.

The sum of proteins per cell predicted from the curated dataset is about 53 million proteins for a fast-growing haploid yeast cell, with 90% confidence limits of 30–80 million. From the local loss-rates for individual proteins through degradation, the global loss-rate through cell growth, and the local protein abundance data, protein synthesis rates can be calculated for each gene. The cell-wide sum of these rates amounts to about 13,000 (6,500–19,500) proteins synthesised per cell per second on average throughout the cell cycle, and, if translation initiation and termination are assumed to be loss-free processes, there must consequently also be about 13,000 translation initiation and translation termination events per cell per second. The frequency of translation elongation events can be calculated locally for each gene if the relevant ORF lengths are included in the calculations, the sum of these amounts to 6.0 (3–9) million elongation events per second per cell.

Interestingly, just under half of this activity (6,400 proteins per second) is required to balance protein degradation, whereas the remainder is required to balance protein dilution through cell growth. One prediction from these results is that protein synthesis is likely to be strongly correlated with cell growth rates, because even if protein decay rates are assumed to be unaffected by reductions in growth rates, the contribution of dilution to the requirements for protein synthesis would decline linearly with such reductions. Experimental evidence supporting these analyses is available from early studies demonstrating that global amino acid incorporation rates, synthesis rates of individual proteins, and translation elongation rates are indeed nearly linearly correlated with growth rates [25,29,33,34]. Moreover, experimentally induced limitations in translational activity produce corresponding growth limitations that are linearly dependent on the former [35].

### The relation between protein synthesis rates and active ribosome numbers

Although predictions of the cellular protein and mRNA content made from the genome-wide datasets are consistent with available experimental data, the quality of predictions of translational activity can be further evaluated by comparison with published evidence relating to the activity of factors participating in the translational process. One way of validating the predicted protein synthesis frequencies is to ask whether the predicted ribosome content of the cell actually matches predicted protein synthesis rates. The proportion of active ribosomes in yeast cells under fast growth conditions has been addressed in independent studies, and these studies consistently report values of around 90% active ribosomes [25,34].

The number of active ribosomes required for the synthesis of an individual gene is a function of protein synthesis rates, ORF length, and translation elongation rates. Under constant translation initiation rates, the numbers of ribosomes that are active under steady-state conditions declines as translation elongation rates increase, essentially because with faster elongation rates ribosomes finish translation sooner and thus become available for further rounds of translation more rapidly.

**Figure 6:**
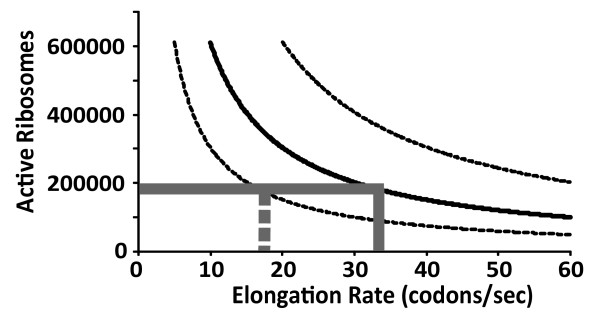
**Calculation of the numbers of active ribosomes as a function of the translation elongation rate**. Local translation rates were calculated for each mRNA from the genome wide expression data as described in the text. The number of ribosomes bound per mRNA was then calculated based on the ORF length and varying translation elongation rates; the sum of all mRNA-bound ribosomes is shown in the graph as a function of the average elongation rate. The solid line was calculated for the mean translational activity (13,000 proteins per cell per second), broken lines were calculated at the upper and lower 90% confidence limit for this parameter (19,500 and 6,500 proteins per cell per second, respectively). Grey lines indicate elongation rates matching a total cell population of 186,000 active ribosomes.

The calculated sum of active ribosomes required for synthesis of all expressed genes is plotted as a function of average elongation rates in figure 6. For protein synthesis frequencies calculated from the curated dataset, the mean value of 186,000 active ribosomes predicted from ribosomal protein and rRNA abundance data is met at an average elongation rate of 32.6 codons per second. There are two published studies available that have addressed translation elongation rates *in vivo *by direct experimental approaches, and these studies reported rates of around 10 codons per second [28,34]. Protein synthesis frequencies predicted from the genome-wide datasets thus appear to require a higher elongation rate than the experimental estimates suggest. However, if the ribosome activity calculations are repeated at the lower limit of the 90% confidence interval for protein synthesis rates (corresponding to 6,500 proteins per second per cell), the ribosomal content is met at an elongation rate of 16.3 codons per second, much closer to the experimental estimate.

It should be noted that the experimental studies in question employed a metabolic labelling technique which required growing the cells in minimal medium, and that cell growth rates were therefore significantly lower than those of cells used for generation of the genome-wide datasets. Since other studies have concluded that elongation rates vary with growth rates (see e.g. ref. [25]), the published value of 10 codons per second can only be taken as a lower limit for elongation rates to be expected under the fast growth conditions used to generate the genome-wide datasets. In conclusion, the relationship between protein synthesis rates, ribosome content and translation elongation rates suggested by the genome-wide datasets appears relatively consistent with experimental estimates, although the comparison with elongation rates may suggest that the true value of intracellular protein synthesis rates lies at the lower end of the confidence interval for this parameter.

### Analyses of nucleotide exchange rates

**Figure 7:**
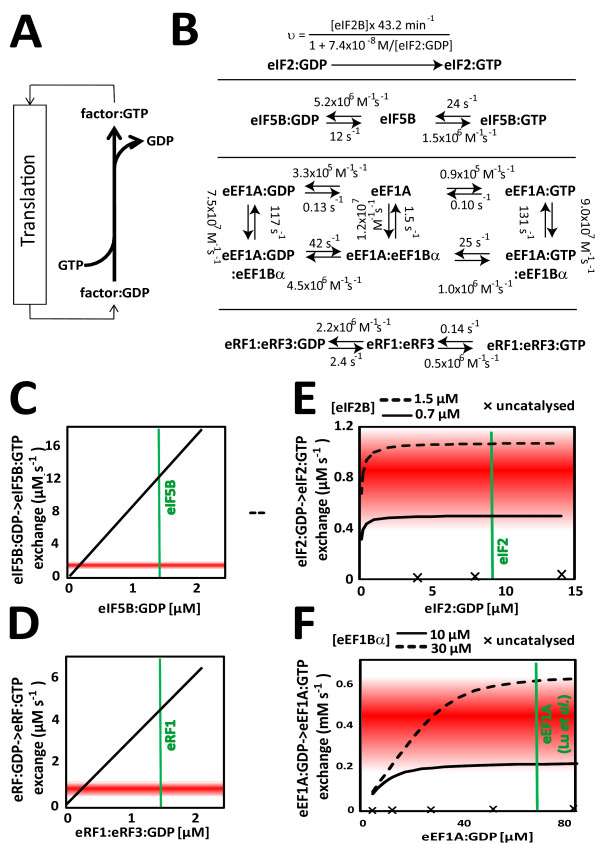
**Analysis of nucleotide exchange activity for different translation factors**. A, nucleotide exchange rates must match protein production rates (for translation initiation and termination factors) or elongation rates (for elongation factors) due to the cyclical nature of the relevant reactions. B, kinetic models used for the analyses in panels C-F. Nucleotide exchange on eIF2 was calculated from published *K*_*M *_(7.4 × 10^-8 ^M) and *k*_*cat *_(43 s^-1^) values according to the Michaelis-Menten equation. All other reactions were implemented as kinetic computer models and analysed for maximum nucleotide exchange rates. Rate constants for the exchange reactions on eIF5B are from [44], on eRF1:eRF3 complexes from [38], and on eEF1A from [8]. C-F results of simulations of nucleotide exchange reactions. In all four panels, areas shaded in red indicate the exchange activity required to match the predicted translational activity, within the 90% confidence limits for these predictions. Nucleotide exchange rates are plotted as a function of the relevant factor:GDP complex concentration (black lines), mean intracellular concentrations of total factor are indicated by green vertical lines for reference (except for eEF1A where the concentration is from a single study, see main text for explanation).

As a further means of verification, published biochemical data on translation factor activities can be used to validate protein synthesis rates predicted from the genome wide datasets. All reactions involving translation factors are of a cyclical nature, where the relevant factors undergo particular reaction steps and then need to be re-set to some initial state in order to catalyse subsequent cycles. It is thus evident that the flux for any given reaction within the translational pathway must equal the total protein synthesis flux, at least if translational reactions are assumed to be 100% efficient and do not occur in futile cycles. Unfortunately, current knowledge on the kinetics of the entire translation initiation, elongation or termination pathways is not sufficiently detailed to predict fluxes from factor levels in the cell. However, several reactions within the translation pathways include the hydrolysis of ATP or GTP, and following these hydrolysis steps the relevant factor:NDP complexes need to be recycled to factor:NTP complexes before the next reaction can occur. Again, the flux through these "mini-cycles" needs to be equivalent to the protein synthesis flux (figure 7A), and for several nucleotide exchange reactions there is sufficient kinetic knowledge available to predict the maximum sustainable flux if intracellular factor concentrations are known.

In yeast, six different translation factors have been identified that hydrolyse nucleotide triphosphates in fixed stoichiometric ratios within translation reactions: these are the initiation factors eIF2 and eIF5B [2], the elongation factors eEF1A, eEF2 and eEF3 [36], and the termination factor eRF3 [37]. Further NTP hydrolysis is required by various helicase activities, although for these it is unknown how many cycles of action are needed per translation cycle. For eIF2, eIF5B, eEF1A and eRF3, relatively detailed data exist on the mechanism and rate constants of the interaction with nucleotides that allow the quantitative estimation of maximum supportable nucleotide exchange rates within the cell (figure 7B). These rates can be usefully compared to the protein production rates predicted from the genome-wide datasets. However, the calculations of nucleotide exchange rates requires factor levels as one of the input parameters, and as has been demonstrated above, available abundance values for individual proteins are associated with a large error. The following calculations therefore have to be interpreted with this caveat in mind.

For eIF5B, exchange of hydrolysed GDP for GTP appears to occur without the involvement of classic guanidine nucleotide exchange factors (GEFs). Exchange rates for this factor are thus a relatively straightforward function of GDP release rates, GTP binding rates, and the GDP-GTP concentration gradient that exists in the cell. Levels of this protein (encoded by the *FUN12 *gene) are equivalent to about 1.4 ± 0.8 μM (average ± SEM of all available data from large- and small-scale studies, calculated for a liquid cell volume of 37 μm^3 ^[12]). Kinetic modelling of the GDP-GTP exchange reaction for this protein as a function of eIF5B:GDP levels shows that maximal rates for this reaction are sufficient to sustain the predicted translation initiation rates upwards of 0.2 μM of the GDP complex (figure 7C). The rate and abundance information available for this protein is thus fully consistent with the predicted translation rates.

For eRF3, the situation is more complicated because GDP exchange rates differ for the free protein and for its complex with eRF1, with the complex being the form of the protein that actually participates in translation termination reactions. For a complete model, rates of eRF3:eRF1 complex formation would thus need to be included, but to date there is no rate information available for this reaction. However, a partial model can be considered that evaluates exchange rates only for the eRF1:eRF3 complex, for which kinetic data are available [38]. According to the genome wide datasets, eRF3 (*SUP35*) occurs in cells in excess over eRF1 (*SUP45*), and we confirmed this excess of eRF3 by independent quantitative western blotting experiments (T. von der Haar and M.F. Tuite, University of Kent, UK, unpublished). Levels of the eRF1:eRF3 complex are thus limited to the abundance of eRF1, or 1.4 ± 0.8 μM. The known rate-constants predict that translation termination rates of 13,000 proteins per second could be sustained with eRF1:eRF3:GDP complex levels as low as 0.3 μM (figure 7D), again indicating that known rate information for GDP exchange on eRF1:eRF3 complexes is fully consistent with the predicted translation rates.

The translation elongation factor eIF2 is one of two known GTPases participating in translation that require guanidine exchange factor activity in order to sustain sufficient nucleotide exchange rates [2]. *K*_*M *_and *k*_*cat *_values have been published for the eIF2B-catalysed nucleotide exchange on eIF2 [5], and these allow the calculation of maximal guanidine exchange rates as a function of eIF2B and eIF2:GDP levels. Intracellular levels of the two factors correspond to 0.74 ± 0.66 μM for eIF2B and 8.7 ± 4.2 μM for eIF2, and at these concentrations maximum guanidine exchange rates can be achieved of 0.6 μM s^-1^. This compares to predicted protein production rates of about 0.4 to 1.2 μM s^-1 ^(calculated as the molar equivalent of 6,500 to 19,500 proteins per second produced in a volume of 37 μm^3^). The *K*_M _value used for these calculations was determined at 20°C, and exchange rates under the experimental conditions used to generate the genome-wide datasets may therefore be marginally higher.

Interestingly, at the assumed factor concentrations, guanidine exchange rates are virtually independent of eIF2:GDP concentrations but instead depend linearly on the concentration of its GEF, eIF2B. This would be consistent with the evolution of eIF2B, rather than eIF2 itself, as the major control point for ternary complex formation in eukaryotes (see e.g. ref. [39]). An increase of eIF2B levels to 1.6 μM would be required to achieve guanidine exchange rates at the upper limit of the 90% confidence interval for translation rate estimates produced here, and it can be concluded that the available kinetic knowledge on eIF2 nucleotide exchange matches translation rates predicted from the genome-wide datasets very closely.

Lastly, nucleotide exchange rates were analysed for the translation elongation factor 1A. Overall nucleotide exchange rates on this factor need to be almost three orders of magnitude higher than for the initiation or termination factors (0.4 mM vs. 0.8 μM), since eEF1A-catalysed GTP hydrolysis occurs once per amino acid bond formed rather than once per protein produced. As for eIF2, guanidine nucleotide exchange on eEF1A is catalysed by an essential GEF, eEF1B. Detailed kinetic models exist for the exchange reaction in the presence of the catalytic subunit of eEF1B, eEF1Bα [8]; and nucleotide exchange rates were therefore here simulated as kinetic models based on these published data. Abundance information for eEF1A, encoded by the identical ORFs *TEF1 *and *TEF2 *in yeast, is particularly variable, with Ghaemmaghami *et al*. reporting 827 molecules per cell (~0.05 μM), while Lu *et al *report 1,181,715 molecules per cell (~70 μM). The available information on the cellular content of eEF1Bα is slightly less variable, with a mean concentration of 15 ± 8 μM. Figure 7F demonstrates that guanidine exchange rates matching the lower limit of the 90% confidence interval for elongation rate predictions (3.3 million amino acid bonds formed per second or 0.2 mM s^-1^) require eEF1Bα concentrations upward of 10 μM and eEF1A concentrations upwards of 30 μM. This would thus be consistent with the higher eEF1A content reported by Lu *et al*.

## Conclusion

Knowledge of the cellular abundance and turnover of gene expression intermediates is an important prerequisite for the quantitative understanding of gene expression. More generally, knowledge of the levels of participating factors is required for the quantitative understanding of any biological process. The analyses conducted in this study show, however, that current datasets reporting such parameters differ widely in the reported values wherever a given gene product is covered by more than one study. These differences are most likely due to errors in the reported values, rather than biological variability under the different experimental conditions. In a recent study by Singh *et al*. [40], a small set of abundance values generated by quantitative western blotting for translation initiation factors was compared to values that had previously been generated in a small scale study in our own work [12], as well as the corresponding values extracted from the large-scale study by Ghaemmaghami *et al*. [14]. Although this study and our previous study employed very different strain backgrounds, the generated data were very linear, indicating an absence of random variability between the two small scale studies. In contrast, although the study by Singh *et al*. and the large scale study both employed relatively closely related (S288C-derived) yeast strains, the comparison between these two studies showed the typical random variation that was also observed here for comparisons between different large scale studies. Random variability thus appears to be a particular hallmark of the large-scale studies, although the reasons for this are currently unclear. It is interesting to note that systematic shifts were also observed in the comparison of the two small-scale studies, and were in fact larger (3-fold) than those observed here between the genome-wide studies.

Whether the extent of error observed in available genome-wide datasets limits their usefulness for quantitative analyses of biological processes essentially depends on the level of detail that needs to be captured. At the level of individual gene products, absolute abundance values (which would for example be important for kinetic modelling or other systems biological analyses) suffer from both the observed random error and the systematic shift error. Simulations of similar datasets indicate that an individual protein abundance value extracted from one of the original genome-wide datasets has associated 50% confidence limits of about 9-fold above and below the reported value, based on a combined random error as observed for the ribosomal proteins in the study by Lu *et al*. [15] and a positioning error as analysed in figure 2. With current datasets, quantitative analyses for individual gene products are thus only reliable in a very broad context. For global parameters however, where the random error element can be predicted to become very small due to averaging effects and only the location error remains, existing datasets permit modelling with higher accuracy.

Based on this principle, this study makes the first available quantitative prediction of global translation rates in logarithmically growing yeast cells. This parameter still has a considerable amount of uncertainty attached to it, mostly because of uncertainty regarding the extent of systematic over- or under-estimation of protein abundance values in the respective studies. It should be noted that in the estimation of confidence limits for translation rates, the protein half-life dataset was assumed not to have a significant location error. This is based on the comparison between the large scale dataset generated by Belle *et al*. and a smaller dataset collated from values reported in the literature, where the distribution of fold different values centres nearly perfectly around one (Figure 1). Because this assumption is based on a single comparison, however, it is not possible to estimate its reliability, and with more datasets becoming available, systematic shifts in protein half-life datasets may yet become apparent.

Another important caveat for the reported activity estimates is that they are based on the assumption that once a ribosome starts translation, a functional protein will always result. Experimental evidence indicates that this is not necessarily the case: for example, a published study on translation in *E. coli *estimated that up to 24% of translation events on an mRNA encoding the *lacZ *protein did not result in synthesis of a functional protein [41]. Because translation occurs co-transcriptionally in *E. coli *and translation initiation can occur on mRNAs that have not yet been completely transcribed, this low proportion of successful translation events is the result of both RNA polymerase and ribosomal failures [41]. In contrast, transcription and translation are uncoupled in eukaryotic cells, and translational failure rates are therefore significantly lower. Investigations of ribosome densities on the yeast *YEF3 *mRNA indicate that processivity failures occured in less than 0.01% of elongation events on this mRNA [42]. In other words, more than 90% of initiating ribosomes resulted in generation of a full-length product on this relatively long mRNA (1045 codons). However, Yef3p is a highly expressed translation factor with a high codon adaptation index, and ribosomal drop-off is more likely to occur on codons decoded by rare tRNAs where wait times are longer [43]. In summary, currently available data are not sufficiently detailed to assess the influence of processivity errors in detail, but such errors are not likely to significantly increase the cellular requirement for translation compared to the estimates given here.

The predicted translation rates of 13,000 (6,500–19,500) proteins synthesised per second and of 6 (3–9) million peptide bonds being formed per second will form a useful benchmark against which emerging knowledge on the kinetics of relevant reactions can be interpreted. In this study, initial analyses have focused on guanidine nucleotide exchange reactions that occur during translation. The resulting analyses show that, to the best available knowledge, biochemical data on such reactions coincide very well with the information on translation rates contained within genome-wide expression datasets. For example, both the uncatalysed exchange reactions for eIF5B and the termination factors are easily sustainable at rates required to regenerate sufficient factor:GTP complexes to match the predicted translation rates, whereas those reactions relying on guanidine exchange factor activity clearly could not sustain sufficient rates if they proceeded in an uncatalysed fashion. Moreover, levels of catalysis as judged from published kinetic data and factor levels extracted from the genome-wide datasets place sustainable nucleotide exchange rates at levels that are required to match the predicted translational activity. Together with the analyses on required ribosomal activity, this study thus suggests that data generated over several decades in different labs, and with various approaches including cell biology, classical biochemistry and modern genome-wide approaches, can be combined quantitatively to produce a coherent picture of a central biological process.

## Supplementary Material

Additional file 1 **Excel spreadsheet containing the literature datasets for protein abundance and protein half-lives, and the full curated dataset.** Click here for file
